# Design, development and biomechanical evaluation of a prefabricated anti pronation foot orthosis

**DOI:** 10.1186/1757-1146-5-S1-P22

**Published:** 2012-04-10

**Authors:** Rachel Majumdar, Philip Laxton, Anna Thuesen, Christopher Nester, Barry Richards

**Affiliations:** 1 Centre for Health Sciences Research, University of Salford, Salford, M6 6PU, UK

## Background

Custom made foot orthoses remain the ‘gold standard’ because the orthotic geometry is tailored to each patient’s foot. However, due to their reduced cost, in some contexts there has been an increasing preference for prefabricated orthoses. Research has failed to identify major differences between the two types of orthosis [1-4]. This project aimed to design, develop and evaluate a new anti-pronation foot orthosis. The project was initiated following observations that many prefabricated orthoses failed to incorporate the design principles used in custom made orthoses and the lack of evidence for the effect of prefabricated orthoses on foot pronation.

## Materials and method

The project comprised three stages. In stage 1 (definition of problem) professional, patient, consumer and retail opinions of existing foot orthoses was sought through unstructured interviews. This produced a technical specification for the new orthosis. In stage 2 (development of orthosis), 80 foot casts were rationalised through observation of cast shape and testing of prototype orthoses to identify a ‘model’ foot shape. Bespoke orthotic materials were formulated and tested to compare durability and stiffness to existing orthosis materials. In stage 3 (evaluation), rearfoot inversion/eversion was measured in 30 people walking and running, in a standard shoe, with and without the orthosis. Marker triads were attached to the heel via a shoe aperture, and to the leg.

## Results

**Figure 1:**
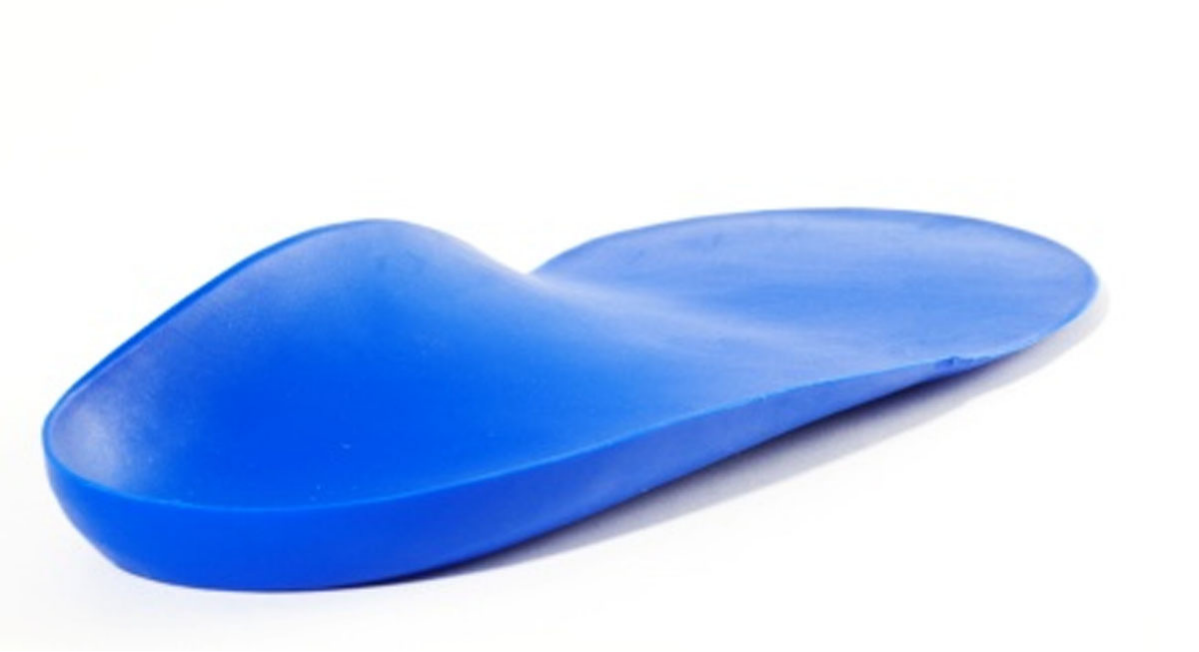
Final orthotic and cross section of arch geometry

**Figure 2:**
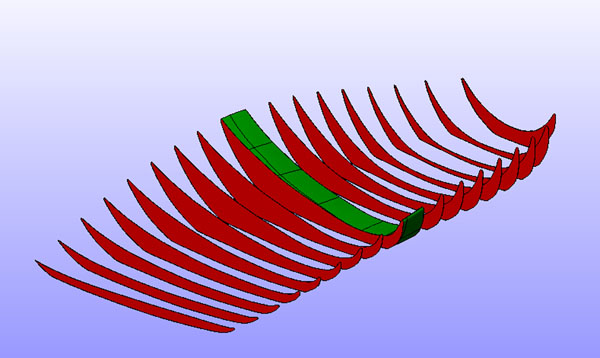
Final orthotic and cross section of arch geometry

**Figure 3:**
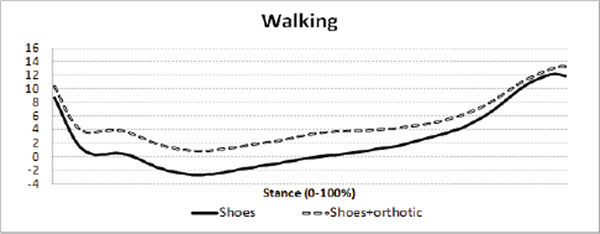
Rearfoot inversion(+ve°) and eversion (-ve°) during walking and running with and without the orthotic. 0° = relaxed standing

**Figure 4:**
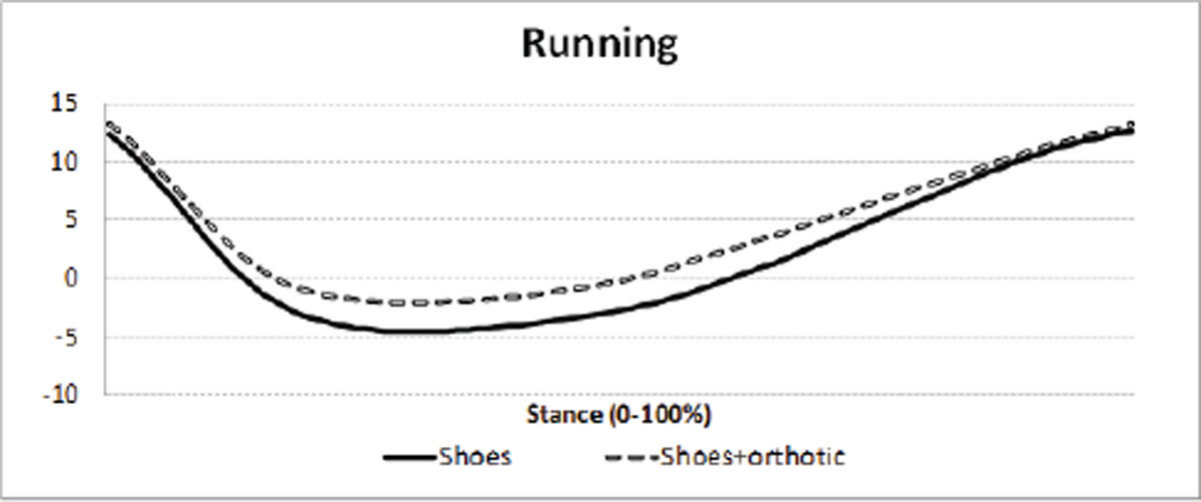
Rearfoot inversion(+ve°) and eversion (-ve°) during walking and running with and without the orthotic. 0° = relaxed standing

The orthotic is illustrated in Figure 1 &2. Maximum rearfoot eversion was reduced (Figure 3 and 4) in both walking (reduced by 3.4°, SD3.5°) and running (by 2.2°, SD2.8°)(p<0.001). The walking reduction is larger than reported by Mills (2.12°) [5] following meta analysis of the literature, but like other reports the orthotic effect was highly person specific.

## Conclusion

The project produced a foot orthosis with evidence of: its design and development process; its material properties compared to existing orthotic materials; its effect on foot pronation.
